# Cardiovascular magnetic resonance T2 mapping detects myocardial edema in patients with chronic dilated cardiomyopathy

**DOI:** 10.1186/1532-429X-14-S1-O29

**Published:** 2012-02-01

**Authors:** Taigang He, Ankur Gulati, Andrew Jabbour, Tevfik F Ismail, Yanqiu Feng, Nizar Ismail, Carla Gonçalves, Tristan Brown, David N Firmin, Sanjay K Prasad, Dudley J Pennell

**Affiliations:** 1 Cardiovascular Biomedical Research Unit, Royal Brompton Hospital, London, UK; 2 National Heart and Lung Institute, Imperial College, London, UK

## Background

Global myocardial edema, a surrogate measure of myocardial inflammation, has been reported in patients with chronic dilated cardiomyopathy (DCM) based on T2-weighted STIR imaging. However, STIR imaging is prone to motion artefact and issues related to cardiac cycle timing and coil sensitivity. We hypothesised that a novel quantitative CMR T2 mapping technique would provide a reliable and reproducible measure for the detection of myocardial edema in chronic non-ischemic DCM.

## Methods

Consecutive DCM patients with disease duration greater than 3 months and age/sex matched healthy volunteers were prospectively enrolled. Exclusion criteria included coronary artery disease, systemic inflammatory disease, acute myocarditis or raised cardiac biomarkers. A mid-ventricular short axis slice was acquired (1.5T MRI scanner; Siemens Avanto, Germany) using a T2-prepared single-shot SSFP readout [1] to generate three T2-weighted images with different T2 preparation times (0 ms, 24 ms, and 55 ms). Parallel imaging was used to reduce the acquisition time and motion-correction applied to facilitate accurate measurement of the T2 times. T2 maps were generated by fitting the exponential decay curve using a linear 2-parameter model after logarithmic transformation. Interobserver variability and interstudy reproducibility were assessed and expressed as coefficient of variation (CoV).

## Results

Ninety DCM (age 52±15 yrs, male 62 (69%), mean LVEF 41%) and 28 healthy volunteers (age 46±12 yrs, male 18 (64%), mean LVEF 68%) were studied. Subjects with DCM had a significantly higher myocardial T2 compared to healthy controls (56.3 ± 3.5 vs 53.7 ±1.7 ms; p<0.001). T2 had good interobserver variability (CoV=1.8%, n=10) and interstudy reproducibility (CoV=1.9%, n=14).

## Conclusions

Quantitative T2 mapping is highly reproducible. Myocardial T2 times are significantly higher in DCM patients than those in healthy controls, suggesting that myocardial inflammation is present in chronic DCM. Further studies are needed to assess the clinical and prognostic impact of these findings in the DCM population.

## Funding

This study was supported by the UK NIHR Cardiovascular Biomedical Research Unit of Royal Brompton Hospital and Imperial College, London. Dr Feng is a visiting academic supported by the National Basic Research Program of China (973 Program) (Grant No. 2010CB732502) and National Natural Science Funds of China (Grant No.30800254). Dr He was supported by Wellcome Trust Value In People (VIP) award and now holds a British Heart Foundation (BHF) Intermediate Basic Science Fellowship (FS/08/26225).

**Figure 1:**
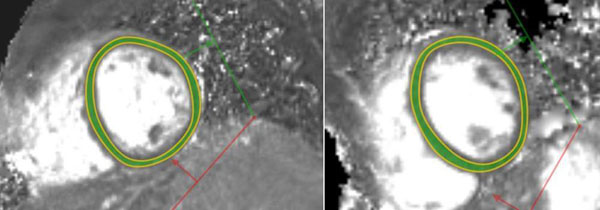
Typical myocardial T2 mappings of a DCM patient (left, T2=59.8ms) and a normal volunteer (right, T2=52.0ms).

**Figure 2:**
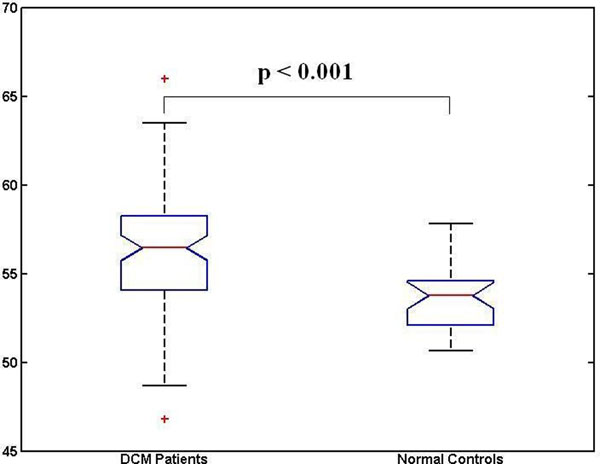
Box and whisker plots of T2 values from DCM patients and normal controls. The boxes have lines at the lower quartile, median, and upper quartile values. The whiskers are lines extending from each end of the boxes to show the extent of the rest of the data.
